# Pro-Reparative Effects of KvLQT1 Potassium Channel Activation in a Mouse Model of Acute Lung Injury Induced by Bleomycin

**DOI:** 10.3390/ijms26157632

**Published:** 2025-08-07

**Authors:** Tom Voisin, Alban Girault, Mélissa Aubin Vega, Émilie Meunier, Jasmine Chebli, Anik Privé, Damien Adam, Emmanuelle Brochiero

**Affiliations:** 1 Centre de Recherche du Centre Hospitalier de l’Université de Montréal (CRCHUM), Montréal, QC H2X 0A9, Canada; tom.voisin@umontreal.ca (T.V.); melissa.aubin.vega.hsj@ssss.gouv.qc.ca (M.A.V.); emilie.meunier908@gmail.com (É.M.); jas.chebli@gmail.com (J.C.); anikprive@gmail.com (A.P.); damien.adam@umontreal.ca (D.A.); 2 Département de Médecine, Université de Montréal, Montréal, QC H3T 1J4, Canada; 3 Laboratoire de Physiologie Cellulaire et Moléculaire (LPCM UR UPJV 4667), 80039 Amiens Cedex, France; alban.girault@u-picardie.fr

**Keywords:** potassium channels, acute lung injury, animal model, pulmonary inflammation, epithelial injury and repair, alveolar–capillary barrier

## Abstract

Acute Respiratory Distress Syndrome (ARDS) is a complex and devastating form of respiratory failure, with high mortality rates, for which there is no pharmacological treatment. The acute exudative phase of ARDS is characterized by severe damage to the alveolar–capillary barrier, infiltration of protein-rich fluid into the lungs, neutrophil recruitment, and high levels of inflammatory mediators. Rapid resolution of this reversible acute phase, with efficient restoration of alveolar functional integrity, is essential before the establishment of irreversible fibrosis and respiratory failure. Several lines of in vitro and in vivo evidence support the involvement of potassium (K^+^) channels—particularly KvLQT1, expressed in alveolar cells—in key cellular mechanisms for ARDS resolution, by promoting alveolar fluid clearance and epithelial repair processes. The aim of our study was to investigate whether pharmacological activation of KvLQT1 channels could elicit beneficial effects on ARDS parameters in an animal model of acute lung injury. We used the well-established bleomycin model, which mimics (at day 7) the key features of the exudative phase of ARDS. Our data demonstrate that treatments with the KvLQT1 activator R-L3, delivered to the lungs, failed to improve endothelial permeability and lung edema in bleomycin mice. However, KvLQT1 activation significantly reduced neutrophil recruitment and tended to decrease levels of pro-inflammatory cytokines/chemokines in bronchoalveolar lavages after bleomycin administration. Importantly, R-L3 treatment was associated with significantly lower injury scores, higher levels of alveolar type I (HTI-56, AQP5) and II (pro-SPC) cell markers, and improved alveolar epithelial repair capacity in the presence of bleomycin. Together, these results suggest that the KvLQT1 K^+^ channel may be a potential target for the resolution of the acute phase of ARDS.

## 1. Introduction

Acute respiratory distress syndrome (ARDS) is a critical respiratory condition that occurs in both adults and children and accounts for ~10% of all intensive care unit (ICU) admissions worldwide [1,2]. Despite improvements in the supportive management of ARDS patients over the past decades, particularly with respect to lung protective ventilation, fluid management, prone positioning, and/or venovenous extracorporeal membrane oxygenation (ECMO) [3,4], mortality rates remain unacceptably high (35–45%) [1,5,6,7]. In addition, ARDS survivors experience long-term physical and psychological complications that severely impact their quality of life [4,8,9]. Unfortunately, there is no effective pharmacological treatment for ARDS. The development of novel therapeutic strategies for the effective resolution of ARDS is therefore urgently needed.

Indeed, ARDS is a complex and heterogeneous syndrome that can develop after either a pulmonary/direct (e.g., pneumonia, gastric aspiration, lung transplantation) or extrapulmonary/indirect (e.g., sepsis, pancreatitis, multiple transfusions) insult [1,4,10,11,12] and results in a dynamic biological response that is unique to each patient. Regardless of its etiology, the pathophysiology of ARDS can be defined in three overlapping phases: exudative, proliferative, and fibrotic. The exudative acute phase is characterized by severe alveolar epithelial and/or endothelial damage, favoring the infiltration of protein-rich fluid into the alveoli, an exaggerated inflammatory response with massive neutrophil infiltration and elevated cytokine/chemokine levels, and impaired lung compliance [4,12,13]. A collagen deposition and fibroproliferation phase, competing with adequate epithelial repair, can rapidly arise and progress to an irreversible fibrotic phase [4,12,13]. Therefore, a therapeutic strategy that can rapidly resolve the parameters of the acute exudative phase and efficiently repair the alveoli prior to the establishment of fibrosis is of critical importance.

Given the major role of inflammation in the pathophysiology of ARDS, anti-inflammatory strategies have been extensively investigated, using both experimental studies (animal models of acute lung injury [ALI]) conducted by our group and others, and clinical trials, with conflicting results [14,15,16,17,18,19,20,21,22,23,24,25,26,27]. It is now becoming increasingly clear that the heterogeneity of ARDS phenotypes contributes significantly to the lack of efficacy of most treatments in clinical studies [14,28,29,30,31,32]. Furthermore, therapies targeting only one aspect of the syndrome, e.g., aimed at controlling only the inflammatory response or improving fluid clearance or surfactant production, often prove insufficient for complete resolution of ARDS [20,26,33,34,35], possibly due to residual alveolar damage. Indeed, the effective restoration of a cohesive and functional alveolar epithelium is critical to promoting ARDS resolution [4,36,37]. Alveolar repair after injury relies on alveolar epithelial type II progenitor cells (ATII) [38,39], which have the ability to dedifferentiate, migrate, proliferate, and then redifferentiate into ATI and ATII cells to recover alveolar integrity and functionality necessary for lung homeostasis. ATII cells are also responsible for surfactant production, which is essential for alveolar stability, while both ATI and ATII cells control sodium (Na^+^) and fluid absorption [40], which is required to maintain almost fluid-free alveolar spaces for efficient gas exchange through ATI cells.

Potassium (K^+^) channels, a large number of which are expressed in the respiratory epithelium, play a key role in several functions of the alveolar epithelium [41,42]. Our previous in vitro studies using primary cultures of alveolar epithelial cells showed that KvLQT1 and K_ATP_ channels, which contribute to a major part of basolateral K^+^ currents, are involved in the process of Na^+^ and—secondarily—liquid absorption through alveolar epithelial cell cultures [43,44,45]. Our data also showed that KvLQT1 channel activation with R-L3 favored the resorption of thiourea-induced lung edema in mice [46]. Similarly, it was previously reported that a K_ATP_ channel opener improved fluid absorption across the alveolar epithelium in human resected lungs [47]. There is also compelling evidence that several types of K^+^ channels are involved in epithelial repair processes [42]. We have shown that the healing of respiratory epithelia, as well as cell migration and proliferation, are dependent on K^+^ channels [48,49,50,51,52], in particular KvLQT1 and K_ATP_. K^+^ channel function has also been investigated in some animal models of ALI. For instance, pharmacological activation of the two-pore domain (K_2_P) K^+^ channel TREK-1 efficiently improved lung injury scores, lung compliance, and inflammatory levels in hyperoxia- and influenza A-induced mouse models, while K_ATP_ activation provided pulmonary protection against warm ischemia-reperfusion injury in rabbit lungs [53,54,55]. Conversely, knockout (KO) of the regulatory subunit (KCNE2) of the KvLQT1 channel in mice has been shown not only to impair the protective response to ischemia-reperfusion injury but also to increase the levels of inflammatory mediators and alter lung function at baseline [56]. We have also recently reported that the full (systemic) KO of the pore-forming (KCNQ1) subunit of the KvLQT1 channel (in KCNQ1^−/−^ mice) alters the inflammatory immune cell profile, exacerbates the negative effect of bleomycin on lung function, and reduces the repair capacity of ATII cells [57]. However, to our knowledge, the effect of the pharmacological activation of KvLQT1 (i.e., KvLQT1 activator delivered directly to the lung) on the resolution of ARDS parameters has never been investigated in an animal model of ALI.

**Figure 1:**
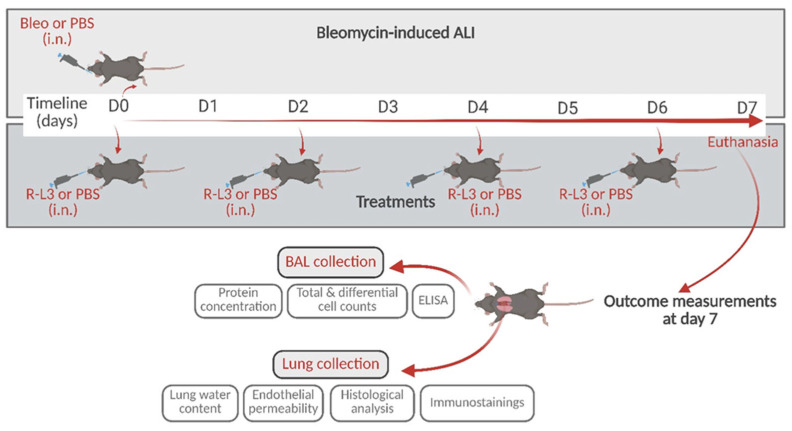
Schematic representation of the experimental design with the time points for bleomycin instillation and treatments with the KvLQT1 activator by intranasal instillation, and subsequent outcome measurements. i.n.: intranasal. Created with BioRender (https://www.biorender.com, accessed on 23 May 2025).

Based on our previous work and data from the literature, we hypothesized that the pharmacological activation of KvLQT1 channels may have beneficial effects on several parameters of the exudative phase of ARDS/ALI. To test this hypothesis, we used the well-characterized model of ALI induced by bleomycin [21,22,57,58,59,60], which mimics key characteristics of the acute phase of ARDS at day 7 post-induction, according to the recommendations of the American Thoracic Society [60,61] (i.e., lung injury, edema, inflammation, and respiratory dysfunction). Treatments with the KvLQT1 activator R-L3, delivered to the lungs, were repeated every two days until day 7 post-bleomycin, when alveolar–capillary barrier function, inflammatory response, lung epithelial damage, and alveolar integrity markers were assessed (see Figure 1 of the schematic experimental design). The repair capacity of the alveolar epithelium was also evaluated in vitro. Our data show that KvLQT1 activation ameliorates the inflammatory state, lung damage, and repair capacity after bleomycin exposure.

## 2. Results

### 2.1. Alterations of the Alveolar-Capillary Barrier After Acute Lung Injury Induced by Bleomycin

**Figure 2:**
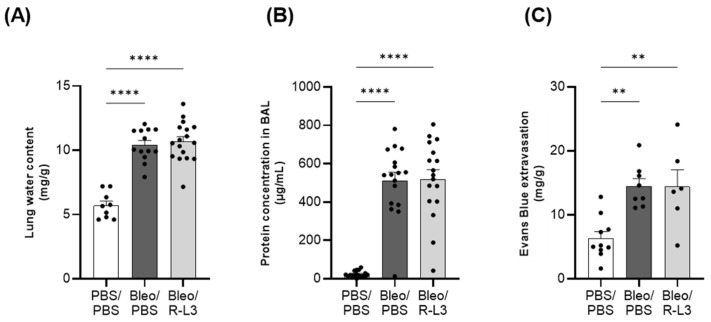
Alteration of the alveolar–capillary barrier after bleomycin-induced acute lung injury. Mice were challenged or not (PBS) with bleomycin (Bleo, 3 U/kg, 50 µL, i.n. on day 0), and the effect of KvLQT1 activation was assessed in mice treated with R-L3 (4 µM, 50 µL, i.n., every 2 days (0, 2, 4, and 6)). On day 7, lung water content (**A**), total protein content in broncho–alveolar lavage (BAL) (**B**), and Evans blue (20 mg/kg, 100 µL, i.v.) extravasation from the circulation to the lungs (**C**) were compared across the 3 experimental groups (PBS/PBS, Bleo/PBS and Bleo/R-L3) (*n* = 9–17). Each point represents one mouse, and values are mean ± SEM. One-way ANOVA (Agostino/Pearson normality test: positive) was performed for (**A**–**C**). ** *p* < 0.01, **** *p* < 0.0001.

We first evaluated the effect of the pharmacological activation of KvLQT1 channels with R-L3 on pulmonary edema flooding during the acute/exudative phase (at day 7) of bleomycin-induced ALI. Three parameters representative of alveolar–capillary barrier integrity and permeability were investigated (Figure 2) by measuring lung water content (A) and total protein concentration in broncho–alveolar lavage (BAL, B) (as indices of protein-rich pulmonary edema) as well as Evans blue extravasation from the circulation into the lung compartment (as index of endothelial permeability, C). As expected, lung delivery of bleomycin was associated with statistically significant increases in lung water content and protein concentration in BAL, indicating the infiltration of protein-rich edema, which was not reduced by the treatment with the KvLQT1 activator (Figure 2A,B). A rise in pCO_2_, reflecting altered gas exchange secondary to alveolar fluid infiltration, was also noted in mice from the bleomycin groups (Bleo/PBS and Bleo/R-L3, Supplementary Figure S1). We also observed an alteration in endothelial integrity after bleomycin exposure, as indicated by the increased concentration of Evans Blue in the lung compartment, which was not reversed by the R-L3 treatment (Figure 2C).

### 2.2. Beneficial Effect of KvLQT1 Activation on the Inflammatory Response Induced by Bleomycin

The bleomycin-induced inflammatory response was first characterized by defining the immune cell composition in BAL, then by measuring the levels of key ARDS/ALI pro-inflammatory cytokines/chemokines secreted in the BAL of mice under physiological conditions (PBS/PBS) and at day 7 after bleomycin exposure and treatment or not (Bleo/PBS) with R-L3 (Bleo/R-L3).

**Figure 3:**
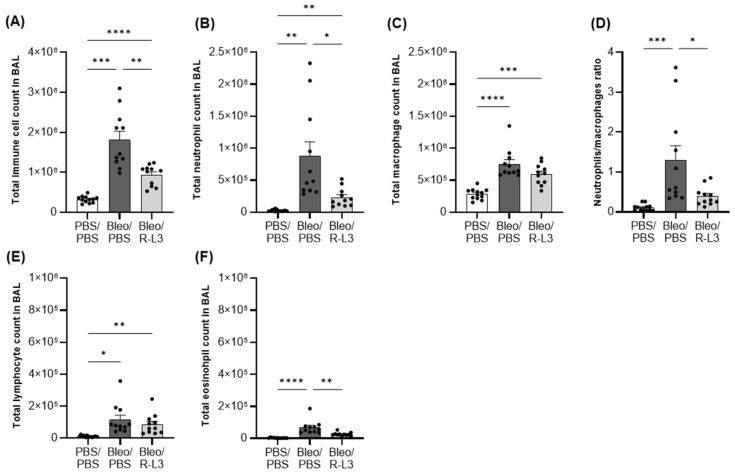
Effect of KvLQT1 activation on the cellular inflammatory response induced by bleomycin. Mice were challenged or not (PBS) with bleomycin (3 U/kg, 50 µL, i.n. on day 0), and the effect of KvLQT1 activation was assessed in mice treated with R-L3 (4 µM, 50 µL, i.n., every 2 days). On day 7, BAL (*n* = 11–12) was collected, and the total immune cell count was determined (**A**). Then, the cell pellet was cytocentrifuged and stained with hematoxylin-eosin to obtain the differential cell counts of neutrophils (**B**), macrophages (**C**), lymphocytes (**E**), and eosinophils (**F**). The neutrophil/macrophage ratio is also reported (**D**). One-way ANOVA test (Agostino/Pearson normality test: positive) was performed for (**A**–**C**). * *p* < 0.05, ** *p* < 0.01, *** *p* < 0.001, **** *p* < 0.0001.

We observed a ~6-fold increase in the total number of live immune cells in BALs after bleomycin exposure (Bleo/PBS group, compared to the control group, PBS/PBS, Figure 3A), mostly secondary to a huge neutrophil infiltration (a known hallmark of ALI/ARDS). To a lesser extent, an increase in the number of macrophages, lymphocytes, and eosinophils was also observed (Figure 3B,C,E,F). Therefore, the neutrophil/macrophage ratio (Figure 3D), a parameter often considered in ARDS, is enhanced 11-fold after bleomycin administration. Interestingly, treatment with the KvLQT1 activator R-L3 significantly reduced the observed rise in total cell number, neutrophil and eosinophil infiltration, as well as the neutrophil/macrophage ratio, after bleomycin challenge.

**Figure 4:**
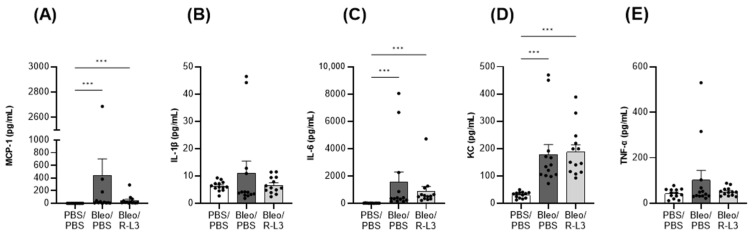
Production of pro-inflammatory mediators after bleomycin challenge. The level of secreted MCP-1 (**A**), IL-1β (**B**), IL-6 (**C**), KC (**D**), and TNF-α (**E**) in BAL, measured by Mesoscale, was compared in the 3 experimental groups (PBS/PBS, Bleo/PBS, and Bleo/R-L3) (*n* = 13). Each point represents one mouse, and values are mean ± SEM. One-way ANOVA test (Agostino/Pearson normality test: positive) was performed. *** *p* < 0.001.

As observed in patients with ARDS featuring either hypo- or hyper-inflammatory profiles [12,31,62,63,64,65], the rise in cytokine/chemokine levels in response to bleomycin-induced ALI was heterogeneous among animals, as shown by multiplex Mesoscale assays (Figure 4). Nevertheless, a significant increase in MCP-1, IL-6, and KC protein levels (as well as a non-significant trend for IL-1β and TNF-α) was measured in BAL from the Bleo/PBS group, compared to the control group (PBS/PBS). KvLQT1 activation (Bleo/R-L3) was associated with a downward trend in MCP-1, IL-1β, IL-6, and TNF-α release in BAL, whereas KC remained unchanged, compared to the Bleo/PBS group.

### 2.3. Beneficial Effect of KvLQT1 Activation on Acute Lung Injury Induced by Bleomycin

**Figure 5:**
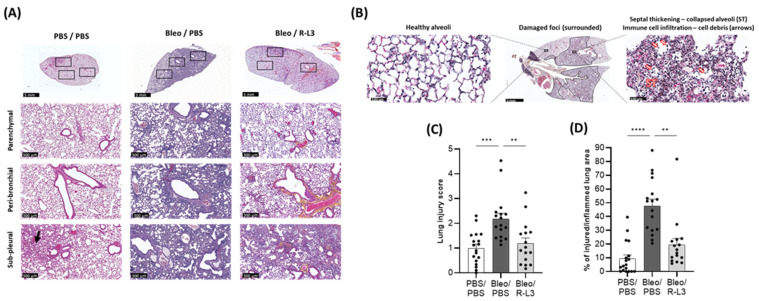
Beneficial effect of KvLQT1 activation on bleomycin-induced injury. Mice were challenged or not (PBS) with bleomycin (3 U/kg, 50 µL, i.n. on day 0), and the effect of KvLQT1 activation was assessed in mice treated with R-L3 (4 µM, 50 µL, i.n., every 2 days). On day 7, lung tissue was harvested and fixed for further hematoxylin-eosin staining. Three representative zones (in parenchymal, peri-bronchial, and sub-pleural regions, **lower panels**, scale: 300 µm) of the whole lung sections (**upper panels**, scale: 3 mm) from each experimental group (PBS/PBS, Bleo/PBS, Bleo/R-L3 at day 7) are shown in (**A**). Enlarged images (**B**) showing both areas with healthy alveoli (**left panel**) and damaged foci (**middle panel**) with septal thickening (ST)/collapsed alveoli, immune cell infiltration, and debris (arrows, **right panel**) are also illustrated. A lung injury score (**C**), taking into account interstitial (septal thickening) and alveolar infiltration (including immune cells and debris), was determined from the whole histological sections (*n* = 16–18 animals per experimental group), using the Visiomorph^®^ software, version 2025.02. A blinded histological analysis of the percentage (%) of injured/inflamed lung areas out of the total lung area of the lung section was also performed (**D**). Each point represents one mouse, and values are mean ± SEM. One-way ANOVA test (Agostino/Pearson normality test: positive) was performed for (**A**,**B**). ** *p* < 0.01, *** *p* < 0.001, **** *p* < 0.0001.

As illustrated in the representative color images of whole sections (Figure 5A, upper panels) and of representative parenchymal, peri-bronchial, and sub-pleural areas (Figure 5A, lower panels), bleomycin-exposed mice (Bleo/PBS column) exhibited dense congestion, cellular (interstitial and alveolar) infiltration of immune cells (macrophage-type mononuclear cells and neutrophil-type polynuclear cells), the presence of debris and/or septal thickening with alveolar structural changes. All these alterations, identified by a team of two pathologists who carried out blinded analyses, are also illustrated in a representative enlarged image (Figure 5B, right panel). Images of the Bleo/PBS group show the heterogeneous nature of bleomycin-induced lung damage within the same lung, with most areas characterized by foci of extensive inflammatory lung damage, juxtaposed with some healthy alveolar zones (Figure 5A,B). The control group (PBS/PBS) exhibited overall healthy alveolar structures with clear alveolar spaces and an absence of injured areas (Figure 5A). Compared to the Bleo/PBS group, mice repeatedly treated with the KvLQT1 activator after bleomycin induction (Bleo/R-L3 group) showed attenuation of septal thickness, immune cell accumulation, and number of damaged foci, resulting in an alveolar architecture more similar to that observed in lung sections from the control group (PBS/PBS). Overall, the injury within the lungs of the Bleo/R-L3 mice appears to be less intense and the damaged areas more scattered than in the bleomycin mice, although some areas still show signs of injury/inflammation.

A quantitative computational analysis of injury scores was performed (Figure 5C) based on the presence of inflammatory (interstitial and alveolar) infiltrate, diffuse alveolar damage with septal thickening, and cellular debris throughout the whole lung sections (including intact and injured alveolar areas to account for the heterogeneous nature of the inflammatory damage) in the three experimental conditions. A significant increase in injury scores was observed in the Bleo/PBS group compared to the basal level in the control group (PBS/PBS). The experimental group treated with R-L3 during the bleomycin challenge (Bleo/R-L3) presented a significant decrease in the injury score, reaching levels similar to those of healthy animals.

We also performed a blinded analysis to define the percentage of injured/inflamed lung area from the total lung section (Figure 5D). Interestingly, KvLQT1 activation with R-L3 in bleomycin-challenged mice (Bleo/R-L3) was associated with a significantly reduced percentage of injured/inflamed areas compared to Bleo/PBS mice.

### 2.4. Assessment of the Alveolar Epithelial Integrity with ATI and ATII Cell Markers

**Figure 6:**
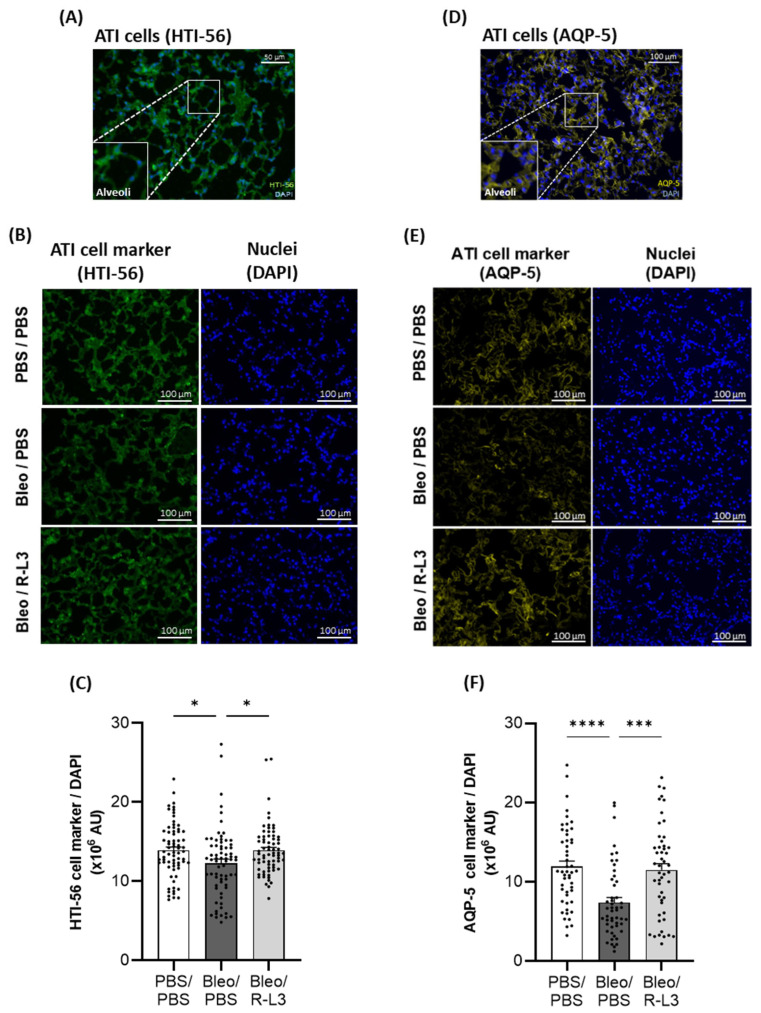
Beneficial effect of KvLQT1 activation on alveolar epithelial (ATI) integrity after bleomycin challenge. Representative color images, with enlarged regional inserts (600×), of merged HTI-56^+^/DAPI (**A**) and AQP5^+^/DAPI (**D**) ATI cell staining of healthy alveoli in lung sections from a control (PBS/PBS) mouse (**A**,**D**). Representative immunofluorescence images of whole lung sections (scale: 100 µm) stained with HTI-56 (**B**) or AQP5 (**E**) from mice treated with R-L3 (4 µM, 50 µL, i.n., every 2 days) or not (PBS) after the bleomycin challenge (3 U/kg, 50 µL, i.n. on day 0). Cell nuclei were stained with DAPI. Quantification (*n*= 50–70 fields) of the pixel intensity of the total HTI-56^+^ (**C**) or AQP5^+^ (**F**) stained surface (covered by ATI cells) was performed in each condition using a protocol exploited by ICY software. Values are mean ± SEM. A one-way ANOVA (Agostino/Pearson normality test: positive) and Kruskal–Wallis test (Agostino/Pearson normality test: negative) were conducted for (**C**,**F**), respectively. * *p* < 0.05, *** *p* < 0.001, **** *p* < 0.0001.

**Figure 7:**
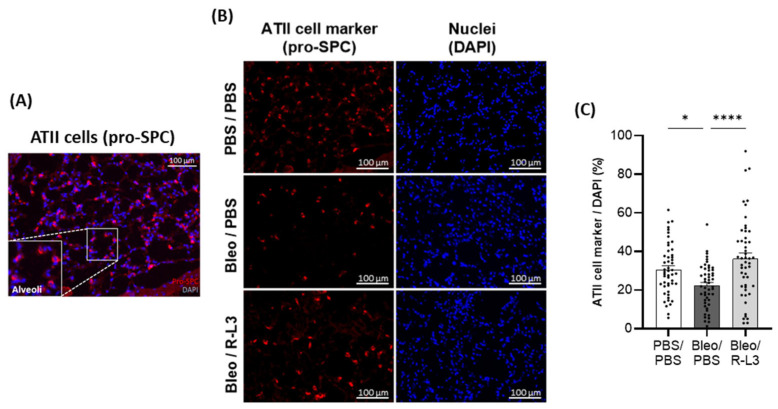
Beneficial effect of KvLQT1 activation on alveolar epithelial (ATII) integrity after bleomycin challenge. Representative color image, with an enlarged regional insert (600×), of merged pro-SPC^+^/DAPI ATII cell staining of healthy alveoli in a lung section from a control (PBS/PBS) mouse (**A**). Representative immunofluorescence images of whole lung sections (scale: 100 µm) stained with pro-SPC^+^ (**B**) from mice treated with R-L3 (4 µM, 50 µL, i.n., every 2 days) or not (PBS) after the bleomycin challenge (3 U/kg, 50 µL, i.n. on day 0). Cell nuclei were stained with DAPI. Quantification (*n* = 50 fields) of the number of pro-SPC+ ATII cells (**C**) was performed in each condition using a protocol exploited by ICY software. Values are mean ± SEM. A one-way ANOVA (Agostino/Pearson normality test: positive) and Kruskal–Wallis test (Agostino/Pearson normality test: negative) were conducted for (**C**). * *p* < 0.05, **** *p* < 0.0001.

To further investigate the effect of bleomycin and R-L3 treatments on alveolar epithelial damage/integrity, immunostaining of specific markers of alveolar type I (HTI-56^+^ and aquaporin 5 (AQP5^+^), Figure 6) and type II (pro-surfactant protein C (SPC^+^), Figure 7) cells were performed (enlarged inserts of stained alveoli are presented in the panels Figure 6A,D and Figure 7A). Our quantitative analyses of the original images of the whole slides (representative images shown in Figure 6B,E) indicate a statistically significant decrease in the intensity of the total surface covered by ATI cells (HTI-56^+^ and AQP5^+^ staining) after bleomycin challenge, which was prevented by the R-L3 treatment (Figure 6C,F). Similarly, KvLQT1 activation counteracted the decrease in ATII (pro-SPC^+^) cell number induced by bleomycin (Figure 7).

### 2.5. Improvement of Wound Healing Repair Rate in Primary Alveolar Epithelial Cell Cultures After KvLQT1 Activation in the Presence of Bleomycin

**Figure 8:**
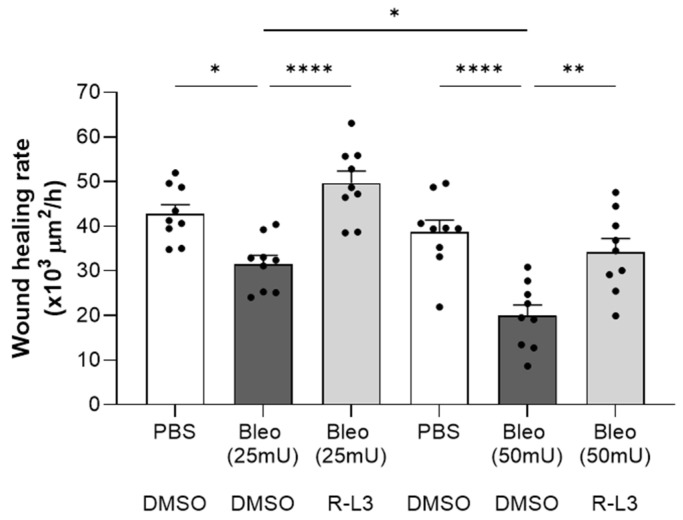
Beneficial effect of KvLQT1 activation on the wound healing rates of primary alveolar epithelial cell monolayers exposed to bleomycin. Wound healing rates after mechanical injury of primary cultures (day 4) of murine alveolar epithelial cells (*n* = 9) were monitored over a period of 24 h and plotted individually (each point representing one alveolar cell culture). Images were taken at t = 0 and t = 24 h to evaluate the wounded area and then calculate the wound healing rates in each of the experimental conditions, i.e., PBS-DMSO (control condition), Bleo-DMSO (at 25 mU or 50 mU), and R-L3 (4 µM) in the presence of bleomycin at 25 or 50 mU (Bleo (25 mU)-R-L3 and Bleo (50 mU)-R-L3). Values are mean ± SEM. A one-way ANOVA (Agostino/Pearson normality test: positive) was performed. * *p* < 0.05, ** *p* < 0.01, **** *p* < 0.0001.

Ultimately, our goal was to evaluate the effects of the damaging exposure to bleomycin and the potential benefit of treatment with R-L3 for the repair capacity of ATII cells, which are progenitors during alveolar repair after injury. Wound healing assays on primary cultured alveolar cell monolayers (Figure 8) showed a dose-dependent inhibitory effect of bleomycin (25 and 50 mU), which was reversed by pre-treatment with R-L3, achieving repair rates similar to the control group (PBS-DMSO).

## 3. Discussion

The aim of our study was to evaluate the effect of KvLQT1 activation, with the pharmacological activator R-L3, delivered directly to the lung, on the main outcomes of bleomycin-induced ALI, during the acute exudative phase (at day 7). As expected, bleomycin instillation into the lung altered the alveolar–capillary barrier (with flooding in protein-rich pulmonary edema), exacerbated inflammatory response (with massive neutrophil infiltration and elevated levels of pro-inflammatory cytokines/chemokines in BAL), and alveolar damage (as indicated by injury scores and a reduction in alveolar epithelial cell markers). Furthermore, bleomycin exposure was associated with reduced repair capacity after injury in primary alveolar cell cultures. Our results also showed that KvLQT1 activation did not improve lung edema resolution and endothelial barrier permeability. However, R-L3 elicited a beneficial effect on the inflammatory response (with significantly reduced neutrophil infiltration and a downward trend of cytokine/chemokine levels in BAL), lower lung injury scores, and preserved alveolar epithelial (ATI, ATII) cell markers in mouse lungs, as well as improved alveolar epithelial repair capacity after injury in vitro.

Measured outcomes on day 7 of the bleomycin challenge confirmed infiltration of a protein-rich fluid (indicated by a significant increase in lung water content and protein concentration in BAL) and alteration of the endothelial barrier (shown by an increase in Evans blue extravasation). Unfortunately, these were not improved by chronic lung treatment with the KvLQT1 activator R-L3. One of our previous studies showed that systemic KO of the KCNQ1 subunit of KvLQT1 did not worsen bleomycin-induced lung edema [57], suggesting that other types of K^+^ channels expressed in the lung [41] act as a compensatory mechanism. In another mouse model of thiourea-induced acute lung edema, R-L3 treatment favored fluid reabsorption at 4 h after thiourea challenge [46]. Similarly, activation of another type of K^+^ channel, i.e., the K_ATP_, attenuated the increase in the wet-to-dry lung weight ratio in a model of ischemia-reperfusion (I/R) injury in isolated rat lungs [66] and favored alveolar fluid clearance in human resected lungs [67]. Differences in protocols, types of damage induced in ALI models, and timing (days for the bleomycin model vs. hours for the thiourea and I/R models) may explain this discrepancy in the benefit, or lack thereof, of K^+^ channel activators. The absence of a protective effect on the endothelial barrier may also be responsible for the persistence of lung edema, despite the treatment with R-L3 in Bleo mice. The mRNA expression of the pore-forming subunit KCNQ1 has been reported in coronary vascular tissue [68]. However, it has not been clearly established whether KvLQT1 channels are expressed in endothelial and/or smooth muscle cells. Indeed, while there is evidence for Kv7 expression in airway smooth cells [69], to our knowledge, the presence of KNCQ1/Kv7.1 channels has not been formally demonstrated in alveolar–capillary endothelial cells. Thus, the lack of KvLQT1 expression/activity in alveolar endothelial cells may explain the failure of R-L3 to improve endothelial permeability and, secondarily, pulmonary edema. The infiltration of fluid into the alveoli of the mice in the Bleo groups, altering gas exchange, was accompanied by an increase in pCO_2_, which has already been reported in bleomycin-treated mice [70,71].

Similar to our previous observations [21,22,57,59], bleomycin-induced ALI was accompanied by a marked increase in the total number of immune cells in the BALs, secondary to massive neutrophil infiltration, a hallmark of ARDS reproduced in this ALI model, and to a lesser extent, an increase in macrophages, lymphocytes, and eosinophils. Importantly, alveolar KvLQT1 activation significantly decreased neutrophil and macrophage infiltration, thereby reducing the total number of immune cells in the BAL. On the contrary, the systemic KO of KCNQ1 did not exacerbate the increase in total immune cells after bleomycin challenge [57].

The beneficial effect of R-L3 on immune cell infiltration, while protein extravasation remained unchanged, is intriguing. This could be explained, as our results suggest, by improvement in the epithelial barrier (preventing interstitial immune cells from infiltrating the alveolar lumen) and/or by a reduction in immune cell chemotaxis. However, further studies are needed to confirm these hypotheses. The benefits of the pharmacological activation of other K^+^ channels for the immune cell response have been reported in several animal ALI models. A decrease in the total cell count in BALs was observed after activation of TREK-1 K^+^ channels in hyperoxia- and influenza A-induced lung injury models [53,54], while treatment with nicorandil, a K_ATP_ channel opener, reduced the levels of myeloperoxidase (MPO), mainly produced by neutrophils, after pulmonary administration of LPS [72]. However, to the best of our knowledge, this is the first observation of a beneficial effect of alveolar KvLQT1 activation on the inflammatory cell response in a bleomycin-induced ALI model.

The Bleo/R-L3 group also exhibited a downward trend in the induction of inflammatory mediators (MCP-1, IL-6, IL1-β, and TNF-α) observed after the bleomycin challenge. Although not statistically significant, such a reduction in the IL-6 and MCP-1 chemokines, which regulate neutrophil and macrophage recruitment, may explain the observed decrease in immune cell counts after R-L3 treatment. A contribution of KvLQT1 channels to the regulation of chemokine secretion would deserve further investigation in future studies. In LPS-challenged mice, K_ATP_ activation with nicorandil significantly reduced TNF-α and IL-1β expression in lung homogenates [72]. On the other hand, TREK-1 activation decreased the hyperoxia-induced IL-6 concentration in BAL (but not TNF-α and CCL-2 (MCP1)) [53], while inhibiting the rise in IL-6 and TNF-α (but not IL-1β and MCP-1) in influenza A-infected mice [54].

Histological analysis of whole lung sections clearly showed lung inflammatory damage in bleomycin mice, which was confirmed by the injury scores and the high percentage of injured/inflamed areas. However, the severity of ALI was heterogeneous among animals in the Bleo/PBS group, as we have previously shown using the same model [22,57]. Importantly, bleomycin mice treated with the KvLQT1 activator showed a significant reduction in injury scores and percentage of injured/inflamed area across the entire section. Consistent with our findings, previous studies demonstrated that the activation of other (K_ATP_ and TREK-1) K^+^ channels counteracted the histological lung injury induced by LPS, hyperoxia, or viral infection [53,54,72]. The histological changes observed in Bleo/R-L3 mice, with a reduced percentage of inflamed areas compared to Bleo/PBS, are also consistent with the decrease in immune cell counts in the BAL of Bleo/R-L3 mice. Further analysis of alveolar epithelial cell markers using the immunostaining of lung sections from the three experimental groups also revealed a decrease in ATI and ATII cell markers in the Bleo/PBS compared to control mice, whereas R-L3 treatment completely reversed the deleterious effect of bleomycin on ATI (HTI-56, AQP5) and ATII (pro-SPC) cell markers, confirming the improvement in alveolar epithelial integrity after lung administration of the KvLQT1 activator. Previous studies of KvLQT1 channel function using ALI models in KO mice have produced conflicting evidence. For example, KO of KCNE2 (which results in decreased KCNQ1 expression) has been shown to lead to increased lung damage at baseline and after ischemia reperfusion [56], while preserved lung integrity has been observed in KCNQ1 KO mice [57]. Differences in protocols, timing, and models could explain these discrepancies.

Finally, we tested a potential beneficial effect of KvLQT1 activation for alveolar epithelial repair in vitro using primary cultures of alveolar epithelial cells. As we previously reported [21], bleomycin exhibited a dose-dependent inhibitory effect on the repair rates after wounding. However, this detrimental impact of bleomycin was reversed by R-L3. Consistent with this result, we previously demonstrated that alveolar cultures from KvLQT1 KO mice exhibited significantly lower healing rates than those from WT mice. These observations are in agreement with previous findings from our laboratory as well as with data from the literature, which revealed the key function of KvLQT1 and other types of K^+^ channels in the regulation of cell migration, proliferation, and epithelial repair [42,48,49,50,51,52,73].

Our repair analyses have focused on epithelial rather than endothelial cell cultures because of the lack of clear evidence for KvLQT1 channels in alveolar capillary endothelial cells and the absence of a beneficial effect of R-L3 on the endothelial barrier. However, the role of KvLQT1 or other K^+^ channel subtypes in the repair of the endothelial barrier deserves further investigation.

In conclusion, our study provides the first proof that alveolar KvLQT1 activation in a bleomycin-induced ALI model improves several parameters (inflammation, injury, and repair). Because of the importance of alveolar damage and repair in the pathophysiology and resolution of the acute/exudative phase of ARDS, the identification of such pro-regenerative strategies is crucial. Further investigation of therapeutic strategies targeting K^+^ channels is however needed, using complementary models of ALI from direct and indirect causes, as well as preclinical human cellular and tissue models, such as primary alveolar cell cultures and living lung tissue (with the protocol of precision-cut lung slices) from ARDS patients, harboring either hypo- or hyper-inflammatory sub-phenotypes. Indeed, patient phenotypes influence not only ARDS outcomes but also the response to treatment, making it difficult to identify effective therapies in models or clinical trials that do not take into account the heterogeneity of the syndrome [14,29,30,31,32]. Therefore, the development of effective precision medicine, adapted to patient phenotypes, constitutes the current challenge in ARDS. Furthermore, complementary approaches, with combined treatments targeting different parameters necessary for full ARDS resolution, may be considered. Pro alveolar repair/regenerative strategies, e.g., targeting K^+^ channels, could be tested in combination with complementary treatment with anti-cytokine drugs (especially for hyper-inflammatory sub-phenotypes of ARDS) or drugs favoring pulmonary edema resolution. Indeed, several ion channels, including other types of K^+^ channels (e.g., K_ATP_ [66,67]) as well as Na^+^ (ENaC) channels, contribute to ion and (secondarily) fluid absorption across alveolar epithelial cells. ENaC channels have also been identified in endothelial cells, and improved alveolar clearance and endothelial barrier have been reported in animal models treated with novel ENaC activators (for review see [74]). Such a strategy could be combined with pro-repair drugs targeting K^+^ channels to improve ARDS resolution. The identification of novel therapeutic targets, including ion channels, is critical in paving the way for effective treatments for ARDS.

## 4. Materials and Methods

### 4.1. Ethical Approval and Animal Care

All animal procedures were approved by the Institutional Committee for the Protection of Animals (approval reference #CM20028EBs/IP19019EBr) of the Centre de Recherche du Centre hospitalier de l’Université de Montréal (CRCHUM) in accordance with the guidelines of the Canadian Council for Animal Care (CCAC).

The C57BL/6 wild-type mice, originally purchased from the Jackson Laboratory, were maintained by breeding them in the CRCHUM animal care facility. The mouse colony was backcrossed every 10 generations. Mice were maintained in a controlled environment with a 12:12 h light/dark cycle and had ad libitum bi to water and food (2018 Teklad global 18% protein rodent diets, Envigo, Indianapolis, IN, USA).

Primary alveolar epithelial cells (see isolation method section below) were isolated from adult male Sprague–Dawley rats (aged 6–7 weeks, weighing 175–200 g) purchased from Charles River Laboratories (Laval, QC, Canada).

### 4.2. In Vivo Experimental Conditions

In vivo experiments were performed on 6–10-week-old mice that were randomly assigned to the experimental groups described below (matched for weight and sex) (Figure 1).

Bleomycin (Bleo, 3 U/kg, Fresenius Kabi, Toronto, ON, Canada) was administered by intranasal instillation (i.n., 50 µL) on day 0 only for ALI induction. The control group received the same volume of PBS (i.n.) on day 0. The effect of the pharmacological activation of KvLQT1 was assessed using intranasal instillation (i.n. of 50 μL) of R-L3 (4 µM, 1/1000 DMSO (vehicle (veh)) in PBS, Tocris Bioscience, Bristol, UK) every 2 days (at day 0 (one hour before bleomycin) and then at days 2, 4, and 6) in the treatment group (Bleo/R-L3). Control mice received PBS (1/1000 DMSO (veh) in PBS, i.n., every 2 days, Bleo/PBS). Therefore, bleomycin and R-L3 compounds were never administered at the same time to avoid potential drug interactions.

The intranasal route of administration was chosen, as previously reported [22,57], because of its non-invasiveness, rapidity (requiring only a brief isoflurane anesthesia from which the animals regain consciousness very quickly), and homogeneous distribution of the administered fluid throughout the lungs after spontaneous nasal aspiration. The dose of 3 U/kg of bleomycin was chosen [22,57] to induce severe lung injury without causing significant mortality. Only a negligible number of mice had to be euthanized because they had reached the endpoints and were therefore excluded from the outcome measurements. The following three experimental groups (i.n. (treatment)/i.n. (challenge)) were then compared: PBS/PBS, Bleo/PBS, and Bleo/R-L3, as indicated in each figure legend. On day 7, mice were euthanized with an overdose of pentobarbital (intraperitoneal injection, i.p.) prior to lung or bronchoalveolar lavage (BAL) collection. Lung tissue samples were used for lung water content assays, Evans Blue extravasation assays and histology/immunofluorescence analyses, while BALs were analyzed to define the inflammatory response and protein levels (Figure 1).

### 4.3. Pulmonary Edema Index

After euthanasia on day 7, the vena cava was cut, and the lungs were removed and weighed (wet weight) [22,46,57,59]. The lungs were then heated at 95 °C for 24 h to measure their dry weight. The water content of the lungs, as an index of pulmonary edema, was then calculated using the following formula [75]:

LWC (mg/g) = (wet weight − dry weight)/mice weight(1)

### 4.4. Evans Blue Extravasation Assay

The Evans Blue extravasation assay, a commonly used technique to evaluate pulmonary permeability after endothelial injury, was performed 7 days after bleomycin challenge or PBS instillation, on mice treated with or without (PBS) KvLQT1 activator (R-L3). Briefly, a solution of Evans Blue (50 mg/kg, Sigma-Aldrich, St-Louis, MO, USA) was injected into the tail vein (intravenous injection, i.v.), and, after 3 h of blood circulation, the mice were euthanized. The lungs were perfused with PBS-EDTA (5 mM, 1 mL) via the pulmonary artery and then washed with successive baths of PBS, as previously described [46,57]. Lungs were minced with scissors and incubated with formamide (Sigma-Aldrich, St-Louis, MO, USA) at 37 °C for 18 h. The resulting homogenate was centrifuged, and the luminescence of the supernatant was measured at 620 and 740 nm to determine the concentration of Evans Blue according to a standard curve. The results were then corrected for heme pigment using the following formula:

E620 (EBD corrected) = E620 − (1.426 × E740 + 0.030)(2)

### 4.5. Analysis of Bronchoalveolar Lavage (BAL) Fluid

In another set of experiments, BALs were obtained after mouse euthanasia on day 7 post-bleomycin challenge using instillation of saline (1 mL) through a catheter [21,22,57,59]. Three replicate BALs (from the same mouse) were pooled on ice prior to centrifugation (700 g, 4 °C, 8 min). Supernatants were aliquoted and stored at −80 °C until they were used to determine protein concentration employing the Bradford method (Bio-Rad Life Science, Mississauga, ON, Canada).

Cell pellets were resuspended in PBS (400 μL) for total immune quantification of live cells (debris was excluded by the use of trypan blue) in a hemocytometer. Cell suspensions were then diluted to a density of 80,000 cells (in 200 μL PBS/slide) before cytocentrifugation (750 rpm, 5 min, Thermo Scientific Cytospin 4 Cytocentrifuge, Block Scientific, NY, USA) onto glass slides and staining with a Hema 3TM Stat Pack (Fisher Healthcare, Waltham, MAUSA). The immune differential cell count (neutrophils, macrophages, lymphocytes, and eosinophils) was then determined.

### 4.6. Cytokine/Chemokine Protein Level Measurements in BAL

Mesoscale multiplex assays for MCP-1, IL-1β, IL-6, KC, and TNF-α proteins were performed on collected BALs [22] according to the manufacturer’s instructions (Mesoscale Discovery, Rockville, MD, USA).

### 4.7. Lung Tissue Collection and Processing

To prevent alveolar collapse, a fixative solution (formalin (500 µL, Chaptec, Montréal, Qc, Canada) or paraformaldehyde (500 µL, Electron microscopy sciences, Hatfield, PA, USA)) was carefully introduced by intratracheal (i.t.) instillation prior to lung harvesting. Tissue samples were then dehydrated before embedding (paraffin (Leica Biosystems, Deer Park, IL, USA) or resin (Shandon Cryomatrix, Thermo Fisher Scientific, Waltham, MA, USA)). Tissue sections (5 μm) were then used either for histological analysis or immunostaining assays.

### 4.8. Histological Analysis

Lung slices were then stained with hematoxylin and eosin (Rapid-Chrome Frozen Sections Staining Kit, Thermo Fisher Scientific, Waltham, MA, USA) and scanned with a Versa stand at 20× objective on a Leica^®^ light microscope, prior to histological analysis by a team of two pathologists or were frozen until immunostaining [22,46,57].

In addition to the qualitative blind histological analysis performed by the pathologists, we also established a lung injury score with computational analysis using Visiomorph™ software (Visiopharm, Hoersholm, Denmark), considering interstitial (septal thickening) and alveolar infiltration (including immune cells and debris), compared to uninflamed healthy alveolar areas. This analysis was performed throughout the lung section to account for the heterogeneous nature of inflammatory damage with both intact and injured/inflamed areas [22,57]. This lung injury score reflects alveolar damage and congestion, altering gas exchange capacity. A blinded histological analysis was also performed to quantify the percentage of lung area with diffuse alveolar damage and immune cell (polynuclear neutrophil) infiltration (expressed as % of injured/inflamed areas of the total area of the lung section) [57]. These lung injury scores were adapted from the official American Thoracic Society workshop report [60,61], a well-established scoring system for experimental models of ALI, and take into account overall (interstitial and alveolar) inflammation, alveolar wall thickening (mainly due to immune cell infiltration), and the presence of debris.

### 4.9. Immunostaining of ATI (HTI56^+^ and AQP-5^+^) and ATII (Pro-SPC^+^) Cells in Cryomatrix-Embedded Frozen Lung Sections

After cryosectioning (5 μm), slides were fixed in 0.4% paraformaldehyde before membrane permeabilization with 0.1% Triton X-100 (specific for pro-SPC immunostaining) and blocked for 1 h with a solution of PBS + 10% FBS (Seradigm, Radnor, PAUSA) + 10% BSA (Sigma-Aldrich, St-Louis, MO, USA). Slides were then incubated overnight at 4 °C with mouse monoclonal anti-HTI-56 (ATI cell, 1:100, #TB-29AHTI-56, Terracebiotech, San Francisco, CA, USA), rabbit polyclonal anti-AQP5 (ATI cell marker, 1:100, # AQP005, Alomone Labs, Jérusalem, Israel), or anti-pro-SPC (ATII cell marker, 1:100, #AB3786 Millipore, Burlington, MA, USA) antibodies. The next day, lung tissues were again blocked before incubation with an Alexa Fluor 633-conjugated donkey anti-mouse secondary antibody (1:200, Sigma-Aldrich, St-Louis, MO, USA) or Alexa Fluor 568-conjugated donkey anti-rabbit secondary antibody (1:200, Life Technologies, Carlsbad, CA, USA) for 1 h, followed by a DAPI nuclear staining (1:1000, Sigma-Aldrich, St-Louis, MO, USA) before mounting with Prolong^®^ Gold (Invitrogen, Thermo Fisher Scientific, Waltham, MA, USA) [46,57]. Images were captured using an Exiqua camera (QImaging, Surrey, BC, Canada) under an inverted fluorescence microscope (Olympus, Richmond Hill, ON, Canada) at 200x (NA = 0.75) and analyzed using ICY software (version 2.4.2.0, license GPLv3, developed by Bio Image Analysis, Institut Pasteur and France-BioImaging, Paris, France) [76]. Our analysis protocol enables the quantitative measurement of the intensity of the total stained surface of the HTI-56^+^ and AQP5^+^ immunostaining (representing the area covered by flattened ATI cells) as well as the number of pro-SPC^+^ regions of interest (ROI) (i.e., the number of cuboidal ATII cells), both normalized to the total number of DAPI-positive cells (approximately 30,000 cells were analyzed for each staining). Our controls showed no background, confirming the specificity of all primary and secondary antibodies used in our assays [46,57].

### 4.10. Isolation and Primary Culture of Rat Alveolar Epithelial Type II (ATII) Cells

ATII cells were isolated from rat lungs according to a well-established protocol [44,45,46,48,57,77,78]. Briefly, lungs were washed with physiological solution to remove excess blood cells and most of the alveolar macrophages. They were then digested with elastase (160 U/lung, Worthington Biochemical, Lakewood, NJ, USA, 30–45 min) in the presence of DNAse I (Roche, Indianapolis, IN, USA). The lungs were then minced, and the resulting cell suspension was filtered. Alveolar epithelial cells were then purified using a differential adherence technique [79], which allows for the discarding of remaining macrophages and fibroblasts attached to IgG-coated Petri dishes and the subsequent collection of an epithelial cell suspension, enriched for up to 86% of ATII cells [43,44,77,78]. Trypan blue staining of the post-IgG cell suspension confirmed >90% cell viability. After counting, the cell pellet was resuspended in MEM (Gibco, Life Technologies Inc., Burlington, ON, Canada) containing 10% FBS (Gibco) supplemented with 0.08 mg/L gentamicin, Septra (3 μg/mL trimethoprim + 17 μg/mL sulfamethoxazole), 0.2% NaHCO_3_ (Sigma-Aldrich, Oakville, ON, Canada), 10 mM HEPES (Hyclone, Fisher Scientific Ltd.), and 2 mM L-glutamine (Gibco, Life Technologies Inc.), as described previously [21,48,52]. This freshly isolated cell suspension was seeded onto Petri dishes (35 mm, Corning, Fisher Scientific Ltd., Nepean, ON, Canada) at a density of 0.04 M cells/cm^2,^ and the medium was replaced with MEM-FBS without Septra after 3 days. Using this protocol, we previously showed that by days 3–4 of primary 2D culture, ~75–85% of the cells exhibited an ATII cell phenotype, while 20–25% transdifferentiated from ATII to ATI-like cells [78].

### 4.11. Wound-Healing Assays

Wound-healing assays [49,50,52,80,81] were performed on primary cultured, confluent rat alveolar epithelial cells. Briefly, alveolar cell monolayers were either pretreated or not (PBS + DMSO (veh)) over a 24 h period with R-L3 (4 µM) before exposure or not (veh-PBS) to increasing doses of bleomycin (25 mU and 50 mU). Then, alveolar cell monolayers were mechanically injured (as per six wounds per Petri dish) using a 10 μL pipette tip according to a highly reproducible technique [46,57]. After injury, the monolayers were washed with MEM-FBS to remove detached, injured cells. A mark under the Petri dish allowed us to photograph the wounds at the same location (at time 0 and after 24 h of repair) with a Nikon camera under light microscopy. The rate of wound closure, expressed in μm^2^/h, was calculated using ImageJ software (version 1.54g, National Institutes of Health, Bethesda, MD, USA) from the wound area measured after repair (t = 24 h) compared to the initial wound area (t = 0 h) for each wound. This time point corresponds to approximately 50% of repair, which allows us to observe either stimulatory or inhibitory effects on the repair capacity as a function of the experimental conditions.

### 4.12. Statistical Analyses

Data are presented as dot plots with mean ± standard error of the mean (SEM). Graphs and statistical analyses were performed using GraphPad Prism version 10 for Windows (GraphPad Software, San Diego, CA, USA). Normality tests (Agostino/Pearson) were performed first, followed by the appropriate statistical tests, as described for each figure legend. Differences were considered significant when *p* < 0.05.

## Data Availability

Data supporting the findings of this study will be shared upon request emailed to the corresponding author at emmanuelle.brochiero@umontreal.ca.

## Supplementary Materials

The following supporting information can be downloaded at: https://www.mdpi.com/article/10.3390/ijms26157632/s1.

## Abbreviations

The following abbreviations are used in this manuscript:

ALI	Acute lung injury
AQP5	Aquaporin 5
ARDS	Acute respiratory distress syndrome
ATI	Alveolar type I cells
ATII	Alveolar type II cells
BAL	Bronchoalveolar lavage
DMSO	Dimethyl sulfoxide
ENaC	Epithelial sodium channel
FBS	Fetal bovine serum
IL-1β	Interleukin-1 beta
IL-6	Interleukin 6
KC	Keratinocyte chemoattractant
KvLQT1	Potassium voltage-gated channel subfamily Q member 1
MCP-1	Monocyte chemoattractant protein-1
PBS	Phosphate-buffered saline
pCO_2_	Partial pressure of carbon dioxide
Pro-SPC	Pro-surfactant protein C
TNF-α	Tumor necrosis factor alpha
