# Supplementary material for: Pro-Reparative Effects of KvLQT1 Potassium Channel Activation in a Mouse Model of Acute Lung Injury Induced by Bleomycin
Source: Int J Mol Sci. 2025 Aug 7;26(15):7632. doi: 10.3390/ijms26157632 (PMC12347741; doi:10.3390/ijms26157632)
Supplement: Supplementary file 1 [file ijms-26-07632-s001.zip › ijms-3499384-supplementary.pdf]

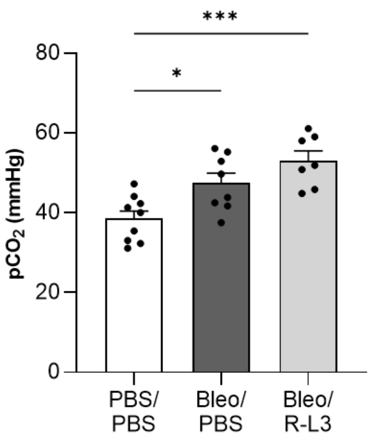

Supplementary Figure S1. Alteration of pCO_2 after acute lung injury induced by bleomycin. Mice were challenged or not (PBS) with bleomycin (Bleo, 3 U/kg, 50 µL, i.n. on day 0) and the effect of KvLQT1 activation was assessed in mice treated with R-L3 (4 µM, 50 µL, i.n., every 2 days). On day 7, pCO_2 values were compared in the 3 experimental groups (PBS/PBS, Bleo/PBS and Bleo/R-L3) (n=7-9). Each point represents one mouse and values are mean ± SEM. One-way ANOVA (Agostino/ Pearson normality test: positive) was performed for A, B, and C. *p < 0.05, ***p < 0.001.
